# Prevalence of liver disease in Colombia between 2009 and 2016

**DOI:** 10.1002/jgh3.12300

**Published:** 2020-02-19

**Authors:** Diana Fernanda Bejarano Ramírez, Gabriel Carrasquilla Gutiérrez, Alexandra Porras Ramírez, Alonso Vera Torres

**Affiliations:** ^1^ Transplant Service University Hospital Fundación Santa Fe de Bogotá Bogotá Colombia; ^2^ Community Medicine and Public Health University El Bosque Bogotá Colombia; ^3^ Department of Public Health University Hospital Fundación Santa Fe de Bogotá Bogotá Colombia

**Keywords:** alcohol consumption, Colombia, immunization, liver disease, obesity, prevalence, unsatisfied basic needs

## Abstract

**Background and Aim:**

Liver disease refers to a set of pathologies resulting from the interruption of liver function or the poor functioning of the liver. The estimation of morbidity and mortality due to liver disease and the context in which the disease develops are determining factors for public policies related to liver disease and its causes. The primary etiologies are cirrhosis and hepatocellular carcinoma, which are directly related to hepatitis B and C virus and alcohol consumption. Followed by hepatotoxic drug use, autoimmune hepatitis, cholestatic diseases, genetic abnormalities, and nonalcoholic steatohepatitis.

**Methods:**

A descriptive cross‐sectional study was conducted to estimate the prevalence of liver disease in Colombia between 2009 and 2016. Using the Data Warehouse–Cube of SISPRO as the primary source of the data, prevalence proportions were calculated and adjusted according to the Bennett Horiuchi method. The relationship with alcohol consumption and the index of unsatisfied basic needs based on estimates from 2005 were considered as sociodemographic variables.

**Results:**

The prevalence of liver disease differs with regard to the type of illness, sex and age of the patient, access to medical attention, and geographical location.

**Conclusions:**

As liver disease is a public health problem, it requires early intervention such as raising awareness and prevention strategies, along with postdiagnosis care channels for treatment, rehabilitation, and palliation. By implementing these strategies, public health will be positively impacted, health care resources will be optimized, and more productive years of life are available for the citizens of the country.

## Graphical abstract

Liver disease is composed of a set of pathologies with nonspecific or without associated symptoms. Between 2009 and 2016 in Colombia, there were 649 887 cases of liver disease. We have shown that prevalence of liver disease is associated to the sales of alcohol reported by departments.

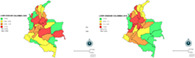

## Introduction

In up to 40% of cases, liver disease is composed of a set of pathologies with either nonspecific symptoms or without associated symptoms.^1^ It has become a significant public health problem due to its high prevalence worldwide, its tendency to progress to advanced stages and even premature death, its resultant decompensation, and its complex and expensive care requirements.^2^, ^3^ Liver disease occurs most frequently in women who mainly suffer from acute liver failure, autoimmune hepatitis, benign liver lesions, primary biliary cirrhosis, hepatotoxicity, and, less regularly, malignant liver tumors, primary sclerosing cholangitis, and viral hepatitis.^4^

Some countries have inaccurate data on the prevalence of liver disease because of underreported numbers of cases and deaths. This underreporting is due to the poor health information tracking systems of these countries.^5^ However, the epidemiological burden of chronic liver disease is increasing due to the increasing prevalence of hepatocellular carcinoma in the terminal stage, nonalcoholic fatty liver disease, and hepatitis C.^1^, ^6^ Viral hepatitis, especially hepatitis B, is the leading cause of cirrhosis in developing countries, while alcohol, hepatitis C, and nonalcoholic steatohepatitis are the major causes of cirrhosis in developed countries; however, other causes such as autoimmune hepatitis, hepatotoxic drugs, cholestatic diseases, genetic abnormalities, and nonalcoholic steatohepatitis can also lead to liver cirrhosis.^1^, ^7^

The occurrence of viral hepatitis is related to specific risk factors such as contaminated food or water intake, blood transfusions, sexual contact between homosexual men, vertical transmission, contaminated hemodialysis equipment, intravenous and intranasal drug use, and so on. In 2015, approximately 257 million people worldwide were living with chronic hepatitis B with the highest prevalence in the WHO African and Western Pacific Regions. Also, 71 million people were living with chronic hepatitis C, mostly in Europe and the Eastern Mediterranean region. Globally, the deaths attributable to viral hepatitis this year approach 1.34 million cases due to chronic liver disease such as cirrhosis (720 000) and hepatocellular carcinoma (470 000) mainly cause by the hepatitis B virus.^8^

In Colombia, in 2012, the vaccination coverage rate against hepatitis B for newborns was 84.72% with an increase in 2016 to 91.25%; however, there were areas with coverage as low as 36–67.4%.^9^

The high consumption of alcohol exponentially increases the risk of cirrhosis^10^ depending on the amount and type of drink (i.e., beer, wine, or alcohol).^11^ A higher risk has been described if consumption is daily or without meals^12^ regardless of the volume ingested.^13^ In 1980, in North America and Australia, there was a decrease in alcohol consumption per capita followed by a reduction of mortality from cirrhosis between 1992 and 2002^14^; in 2010, in Latin America, the average alcohol consumption ranged between 2 and 8 L/year per person, and in Colombia, between 4 and 6 L.^15^ Alcohol is responsible for 5.9% of deaths worldwide and the loss of 139 million years of life adjusted for disability due to cancer, cardiovascular diseases, liver cirrhosis, and depression. It also increases the risk of traffic accidents, self‐inflicted injuries, suicides, falls, and drownings.^16^

The high prevalence of obesity and metabolic syndrome is recognized as a significant cause of morbidity and mortality and generates a higher incidence of cirrhosis secondary to nonalcoholic liver disease, especially in developed countries.^1^ In 2008, nonalcoholic steatohepatitis accounted for 75% of all chronic liver diseases^6^; however, although it typically has a benign course, it can progress to cirrhosis in 25% of cases and cause deaths in 10% of cases—severe liver disease.^17^

Metabolic diseases usually manifest in childhood, and their advanced stages require liver transplantation. In the United States, liver transplants due to metabolic diseases represent approximately 4% of all liver transplants in adults and 20% of those in children (mainly due to α1‐antitrypsin deficiency, amyloid polyneuropathy, hereditary hemochromatosis, and cystic fibrosis).^18^

The annual number of cases of liver disease and its resulting complications have a negative impact on the health status of the Colombian population. This is increasing due to people's lack of access to health care as well as socioenvironmental, cultural, economic, educational, genetic, and behavioral factors. These factors constitute a social health disparity^19^ that could potentially be remedied by a political decision in favor of promoting equity of access to health care.

The impacts of liver disease in Colombia have not been reported, which represents a limitation for effective primary care strategies. The primary objective of this study was to estimate the prevalence of liver disease in Colombia between 2009 and 2016 and to evaluate associated factors. The secondary objective was to broaden the epidemiological profile and provide the analysis of socioeconomic inequalities to guide public policy decision‐making by recommending effective intervention strategies at different levels of health care.

## Methods

A list of 139 diagnoses of liver diseases was defined. Twelve study categories were created in conjunction with an expert according to the tenth version of the *International Classification of Diseases* (ICD‐10): cirrhosis, portal hypertension, congenital and at birth liver disease, pregnancy, alcoholic liver disease, noncirrhotic liver disease, inflammation, metabolism, toxicity, tumors, vascular disease, and viral infections. To estimate the prevalence of liver disease in Colombia, the Data Warehouse‐Cube of SISPRO was used as the primary source of data^20^ since it had information from the Individual Registry of Health Service Delivery (RIPS) of the Ministry of Health of Colombia.

The calculation of the prevalence proportion included the variables of sex, date of care, ICD‐10 principal diagnosis category, quinquennial age groups, area of residence, and number of consultations, procedures, hospitalization, and emergencies; this ensured that each person was included only once, even if they received more than one treatment during the defined period.

To calculate the prevalence rate of liver disease per year, we made adjustments by underreporting using the method of Bennett Horiuchi^21^ based on the intercensal estimate of deaths between 2009 and 2016 distributed by sex, department and age group divided by quinquennial per 1000 inhabitants. As a denominator, the population projections of the National Administrative Department of Statistics–DANE^22^ was used for each of the years of study, and the adjustment was made according to population composition with a direct method using the 2005 census population as the reference population. The proportion of population insured by the health system was estimated by year, sex, and disease category and the georeferencing maps were made by year and category of disease.

To analyze whether there was a change in the prevalence rate of general liver disease and its categories, a probability distribution of Poisson occurrence was made, which does not show changes over time. Thus, the Joinpoint regression model for the liver disease in general and, accurately, for liver tumors, cirrhosis, and viral infections was used. The Poisson and Joinpoint regressions were performed based on the adjusted prevalence per 1000 inhabitants in the STATA® (Lakeway Drive, College Station, TX, UU) version 14^23^ and Joinpoint [National Cancer Institute (NCI), Division of Cancer Control of Populations Sciences. Surveillance Research Program, Bethesda, MA, USA] version 4.6.0.0 from April 2018.^24^

The evaluation of inequalities in health was carried out in EPIDAT® (The EpiData Association, Odense M, Denmark, Europe) 4.2^25^ using concentration curves and calculation of inequality indexes based on the annual prevalence proportions and by disease category with the index of unsatisfied basic needs (NBI) corresponding to that calculated for the 2005 census.

For the estimation of the relationship of cirrhotic disease with alcohol consumption, the data reported in the 2012 alcohol sale study and the adjusted prevalence of alcoholic liver disease by areas in Colombia were taken.^26^ The analysis of the relationship between alcohol consumption and liver cirrhosis was made in version 24 of the SPSS® program^27^ from the prevalence data of the cirrhotic disease and the per capita alcohol sale rates of the 2012 population projection using the Kendall correlation in the 2009–2016 period.

## Results

In Colombia, 649 887 people with liver disease were treated between 2009 and 2016, with evidence of an annual increase in the number of cases/year up to 2014 (Table 1).

**Table 1:** Prevalence of liver disease per 1000 inhabitants. Colombia, 2009–2016

Year	Cases	Cases × 1000 inhabitants	Adjusted proportion	95% CI	
2009	55 649	1.244	1.213	1.201	1.216
2010	55 766	1.232	1.184	1.175	1.187
2011	71 825	1.567	1.487	1.476	1.491
2012	86 394	1.863	1.749	1.735	1.761
2013	91 400	1.946	1.811	1.799	1.823
2014	108 242	2.278	2.086	2.073	2.100
2015	91 380	1.902	1.725	1.713	1.729
2016	89 231	1.837	1.640	1.630	1.645

CI, confidence interval.

Further, 58% (378966) of the cases correspond to women and 42% (270921) to men, with higher prevalence seen between 40 and 69 years (Table 2).

**Table 2:** Distribution of cases of liver disease by age groups (decades). Colombia, 2009–2016

Age group	Female (%)	Male (%)	Total
0–9	2	3	31 677
10–19	4	3	45 579
20–29	6	4	65 557
30–39	8	5	84 047
40–49	10	7	112 421
50–59	13	9	140 818
60–69	9	6	101 560
70–79	5	3	50 741
80 and more	2	1	17 487
Total	58	42	649 887

**Figure 1:**
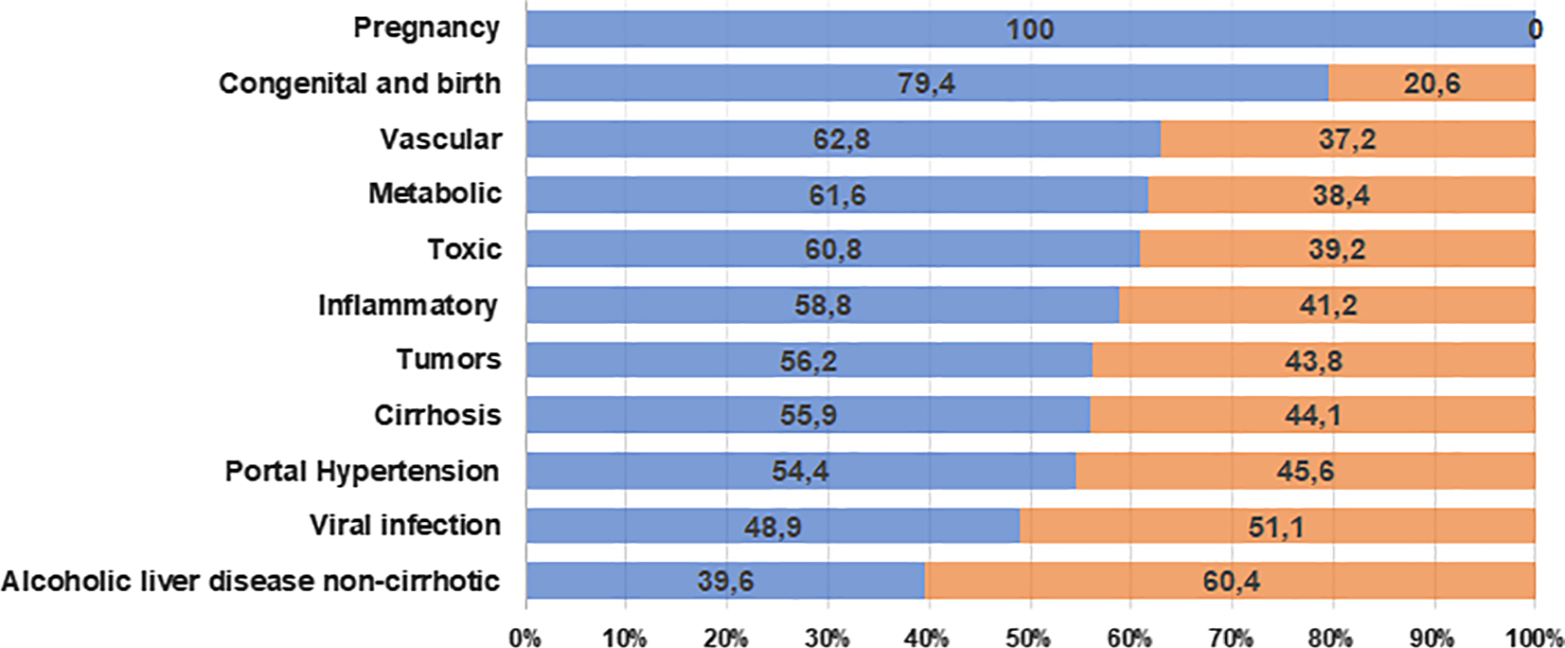
Proportion of cases by category of liver disease by sex. Colombia, 2009–2016. (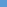) Women; (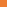) men.

According to disease categories, the highest proportion corresponds to metabolic diseases (55.2%), cirrhosis (20.8%), and viral infections (13.6%), followed by inflammatory diseases (3.4%), liver tumors (3%), and liver toxicity (2.7%); however, vascular diseases, pregnancy, congenital and at birth disease, and portal hypertension had less than 2% of total liver disease, with significant differences by sex (Fig. 1).

### *Prevalence of liver disease*

**Figure 2:**
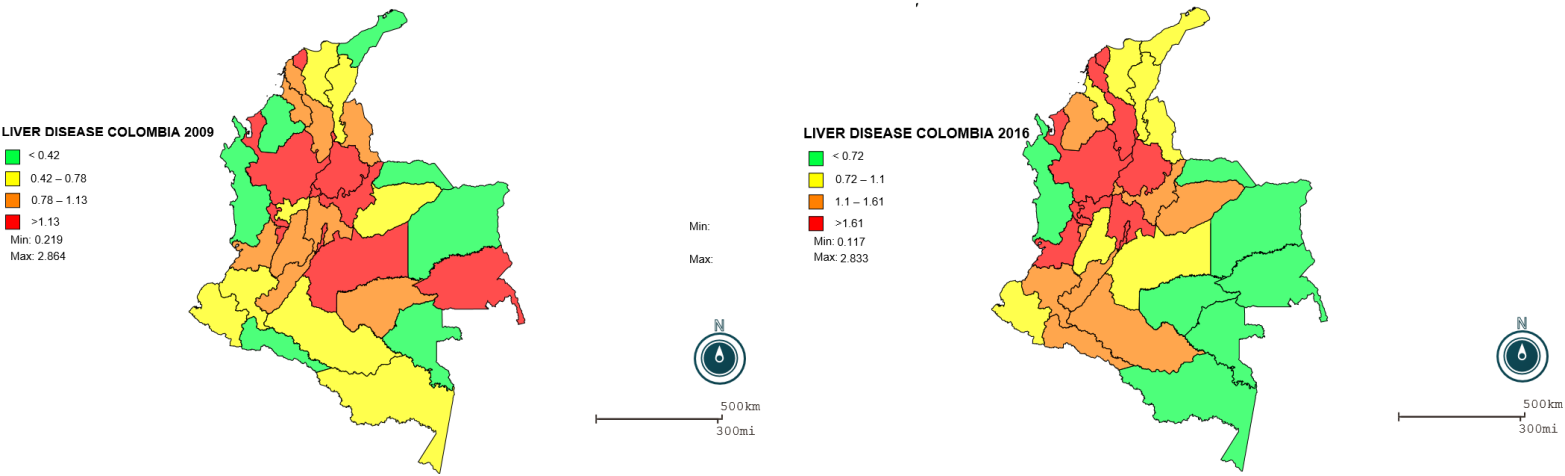
Geographic distribution of liver disease Colombia, 2009 and 2016.

Liver disease caused 55 649 cases in 2009 with 56% of those in in women; the national adjusted proportion was 1.21 × 1000 inhabitants; at departmental (state)‐level prevalence ranged between 0.0218 and 2.8635 × 1000 inhabitants; for 2016, the cases amounted to 89 231, (60% in women), 1.64 × 1000 inhabitants nationwide and 0.1173 and 2.8329 × 1000 departmental (state) inhabitants (Fig. 2).

**Figure 3:**
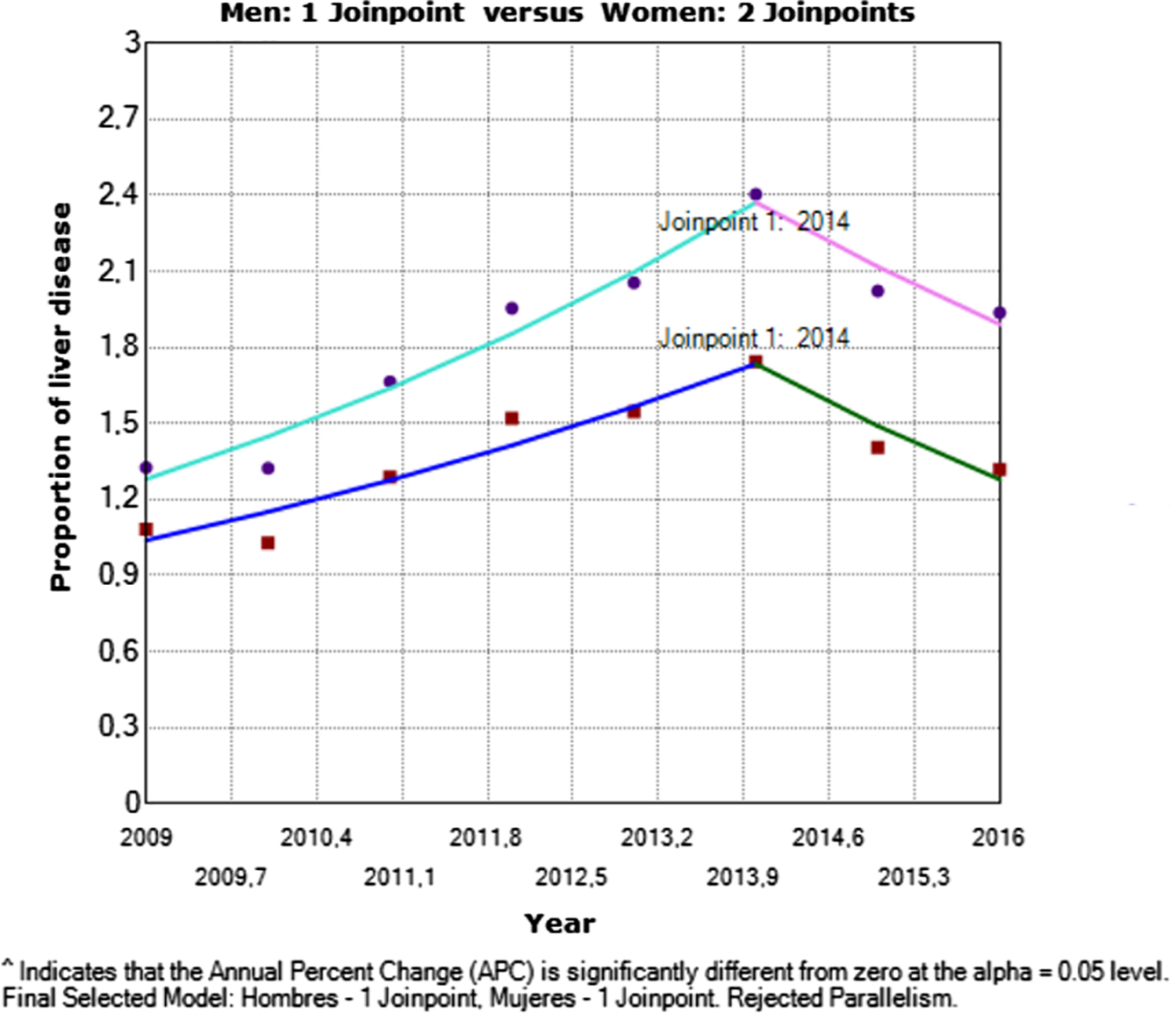
Liver disease in Colombia by sex in the period of 2009–2016. One man *versus* two women. (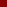) Men; (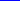) 2009.0‐2014.0 APC = 10.78†; (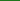) 2014.0‐2016.0 APC = 14.12; (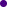) women; (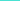) 2009.0‐2014.0 APC = 13.12†; (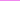) 2014.0‐2016.0 APC = 10.71. †APC is significantly different from zero at the alpha = 0.05 level. Final selected model: men – one joinpoint, women – one joinpoint. Rejected parallelism.

Between 2009 and 2014, there was a significant increase in the number of cases, with an annual change rate of 12.07% (95% confidence interval [CI]: 4.6 to 20.1, *P* = 0.013).The similar straight line gradients with statistical significance (*P* < 0.00) show changes of 10.78% in men (95% CI: 2.5–19.7, *P* = 0.024) and 13.1% in women (95% CI: 6.2 to 20.4, *P* = 0.008) with a significant slope matching test (*P* = 0.0002). This indicates that there were no differences in the prevalence of liver disease by sex in the study period (Fig. 3).

### *Liver cirrhosis*

There were 9650 cases of liver cirrhosis reported in 2009 (52% in women); the adjusted national proportion was 0.20 × 1000 inhabitants, the departmental (state) adjusted proportions ranged between 0.000 and 0.956 × 1000 inhabitants. In 2016, 22 588 cases of liver cirrhosis were reported (59% in women); 0.40 × 1000 inhabitants were affected nationwide and between 0.031 and 0.771 × 1000 inhabitants experienced liver cirrhosis at departmental (state).

**Figure 4:**
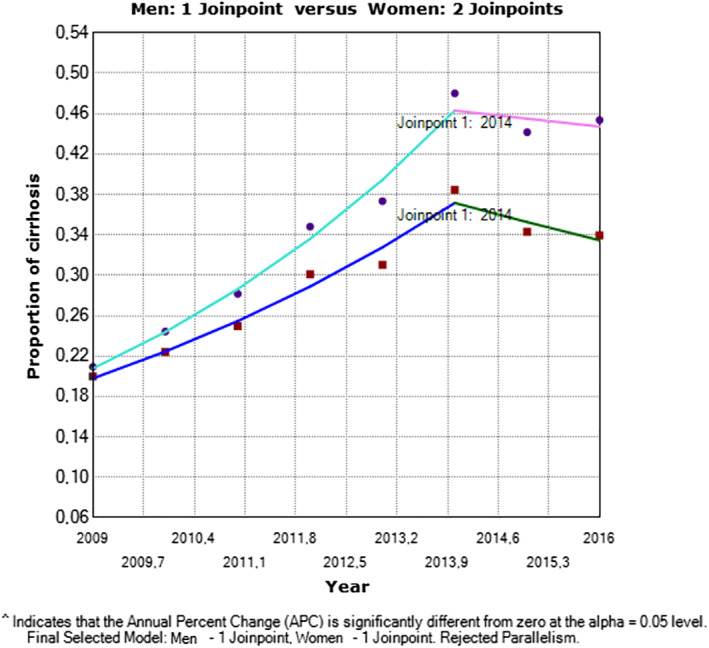
Cirrhosis in Colombia by sex in the period of 2009–2016. One man *versus* two women. (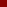) Men; (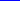) 2009.0‐2014.0 APC = 13.42†; (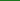) 2014.0‐2016.0 APC = −5.10; (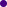) women; (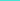) 2009.0‐2014.0 APC = 17.39†; (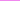) 2014.0‐2016.0 APC = −1.73. †APC is significantly different from zero at the alpha = 0.05 level. Final selected model: men – one joinpoint, women – one joinpoint. Rejected parallelism.

Between 2009 and 2014, hepatic cirrhosis increased significantly by 15.55% (95% CI: 11.0 to 20.3, *P* = 0.0012), with a further decrease until 2016 (APC −0.48% 95% CI: −14.1 to 15.3, *P* = 0.001); the percentage of change in the total range presented significant differences (average annual percent change AAPC 9.9% 95% CI 6.5 to 13.3, *P* < 0.00). By sex, the adjusted proportions are greater in women with 17.39% of annual change (95% CI 12.9 to 22.1, *P* = 0.0006), and men exhibited a change of 13.4% (95% CI: 8.8 to 18.3, *P* = 0.0023). Both sexes presented statistically significant differences until 2014; between 2014 and 2016, the slopes decreased without statistically significant differences (Fig. 4).

### *Metabolic diseases of the liver*

During 2009, 28 743 cases of metabolic disease of the liver were reported, 61% of those in women; the adjusted national proportion was 0.62 × 1000 inhabitants; at the departmental (state) level, the prevalence ranged between 0.000 and 7.149 × 100 inhabitants. For the year 2016, the number of cases increased to 50 836, mostly in women (63%), 0.94 × 1000 inhabitants nationwide and between 0.000 and 12.809 × 1000 inhabitants at the departmental (state) level.

Metabolic diseases showed progressive increase between 2009 and 2014 with 14.63% annual change (95% CI: 3.9–26.5, *P* = 0.02), and a subsequent decrease of 11.72% of annual change (95% CI: −39.8–29.5, *P* = 0.4), the average annual percent change AAPC (6.4%, 95% CI: −1.8–15.3, *P* = 0.1). By sex, women presented higher proportions of metabolic liver disease than men with a similar trend of data: in women, 14.64% annual change (95% CI: 4.4–25.8, *P* = 0.01) and 14.55% in men (95% CI: 2.9–27.5, *P* = 0.02). Statistically significant differences were observed in the first slope and junction, and the parallelism test was significant for both slopes (*P* = 0.013).

### *Primary tumors of the liver*

During 2009, there were 1374 people, 55% of them women, diagnosed with primary tumors of the liver; the adjusted national proportion was 0.029 × 1000 inhabitants; the departmental (state) adjusted proportions ranged between 0.000 and 0.052 × 1000 inhabitants. In 2016, liver tumors represented 2132 cases (56% were women), 0.037 × 1000 inhabitants nationwide had liver tumors, and between 0.000 and 0.059 × 1000 inhabitants at the departmental (state) level also presented with tumors.

Between 2009 and 2013, the slope rises significantly with a 31.39% annual change (95% CI: 17.4 to 47.0, *P* = 0.004), followed by a deceleration from 2013 to 2016 to ‐25.03% annually (95% CI: −36.0 to −12.1, *P* = 0.01). The graph by sex shows similarity between 2009 and 2013, with p values of 0.005 (APC 35.13%, 95% CI: 17.9 to 54.8) in women with liver tumors and 0.004 (APC 27.02%, 95% CI: 15.6 to 39.6) in men with liver tumors. The slopes between 2013 and 2016 presented *P* = 0.012 (APC −27.59%, 95% CI: −40.1 to −12.5) in women and 0.010 (APC −21.7%, 95% CI: −31.7 to −10.3) in men.

### *Viral infections of the liver*

In 2009, 12 124 cases of viral infections of the liver were reported. The adjusted national proportion was 0.28 × 1000 inhabitants, and at the departmental (state) level, it ranged between 0.000 and 0.448 × 1000 inhabitants; by sex, men had a higher prevalence (53%) of viral infections of the liver. However, in 2016, the number of cases decreased to 7439, without significant differences between men and women (i.e. 49 and 51%, respectively).The adjusted national proportion was 0.14 × 1000 inhabitants with viral infections of the liver, and between 0.008 and 0.355 × 1000 inhabitants at the departmental (state) level (Fig. S1, Supporting information).

Viral infections between 2009 and 2016 did not show statistically significant differences in slopes or junctions (APC 2.16%, 95% CI: −10‐9 to 17.2). In 2013, viral infections presented a decrease of 19.6% annual change (95% CI: −37.7 to 3.8), without being significant, as well as showing no statistically significant differences in the average annual percentage change by sex (−7.8%, 95% CI: −15.1 to 0.2, *P* = 0.1).

The disease categories of congenital and birth, portal hypertension, pregnancy, alcoholic non‐cirrhotic liver disease, inflammation, toxicity, and vascular disease had lower prevalence in the study period (Table S1).

### *Sociodemographic variables*

With respect to the relationship between the adjusted prevalence rate of liver disease presenting as cirrhosis and the per capita alcohol sales in Colombia in 2012, the departmental (state) with the highest per capita alcohol consumption showed an increase in the prevalence of the disease between 2012 and 2016 (Fig. S2).

Kendall's correlation coefficient of prevalence proportions and per capita alcohol sales is statistically significant for liver disease in 2012 and 2016 (Kendall's tau coefficient *b* = 0.320, y = 333, *P* = 0.022, *y* = 0.017, respectively), and cirrhosis in 2016 (Kendall's tau coefficient *b* = 0.284, *P* = 0.043).

**Figure 5:**
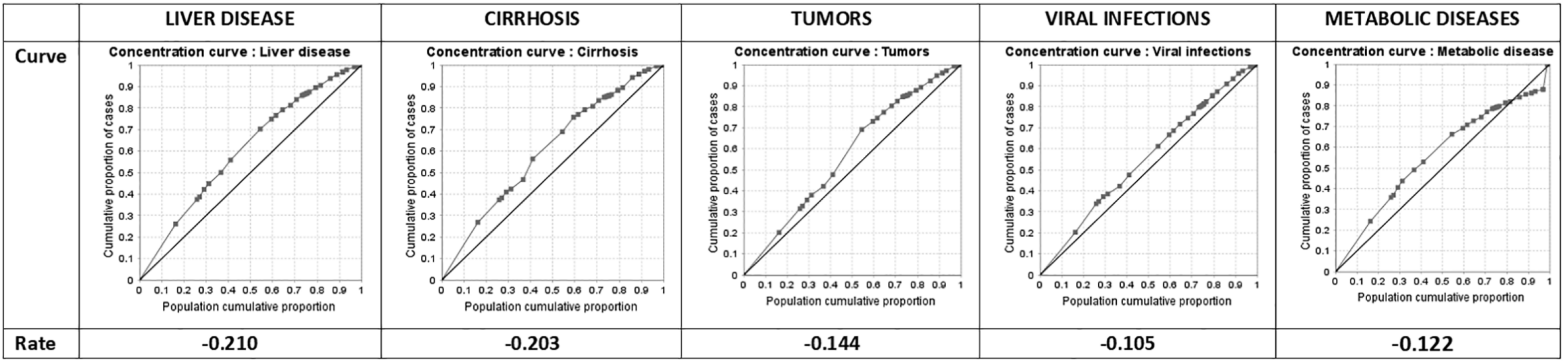
Concentration curves of prevalence according to the index of unsatisfied basic needs.

Fifty percent of the cases of liver disease are concentrated in 38% of the population with the highest NBI (the poorest), and 48% of the cases of cirrhosis occurred in 38% of the most impoverished people. By the same token, 49% of the cases of liver tumors occurred in 42% of the people with the highest NBI, but 30% of viral infections and metabolic diseases occurred in the wealthiest 40% of the population (lowest NBI index) (Fig. 5).

## Discussion

In Colombia, there were 649 887 cases of liver disease between 2009 and 2016. Fifty‐four percent of cases occurred in the age group 40–69 years and 58% corresponded to women. Regarding the categories of disease, differences were found according to geographical location, perhaps due to specific social determinants that should be further explored. However, metabolic disorders, liver cirrhosis, and viral infections had the highest prevalence (55.3, 20.8, and 13.6%, respectively). In general, liver disease, metabolic diseases, and liver cirrhosis showed significant increase between 2009 and 2014.

The distribution of liver disease and cirrhosis occurs worldwide with the most significant differences by geographic location.^1^ Our findings are similar to those described by Escorcia and Marrugo,^28^ with the highest proportion of liver disease found in people older than 60 years (59.9%) and mainly in women (62.3%) with differences in geographical distribution and type of affiliation to insurance of the health system.

We report the occurrence of liver disease associated to socioeconomic factors such as index of NBI and to alcohol consumption. Disparities (i.e. socioeconomic status, age, geographic location, gender, social environment, education, literacy, culture, and behavior) are associated with a large proportion of patients with liver disease as well as higher mortality rates.^19^, ^29^, ^30^

We have shown that prevalence of liver disease is associated with the sales of alcohol reported by departmental (state) in 2016. It was also reported by Andrade and Cols^26^ that areas with higher sales of alcohol per capita are directly impacted by having higher rates of alcoholic liver disease. Alcohol consumption is a risk factor for liver cirrhosis.^10^, ^31^, ^32^

Between 2009 and 2016, 58% of the cases of liver disease in Colombia were women. Higher prevalence in females is mediated by physiopathological processes that favor faster progression of the disease and development of complications.^4^

Chronic liver diseases have a poor prognosis, reduce life expectancy between 20 and 38 years in people aged 30 to 50 years, and have economic impacts in patients and families.^1^ Liver cirrhosis is associated with a substantial economic burden.^33^ In 2004, in the United States, liver disease (liver cirrhosis, liver disease, alcoholic hepatitis, fatty liver) was classified as the most expensive disease, costing a total of 13.1 billion dollars (80.6% indirect costs and 19.3% direct costs).^34^

Our study is one of the first to estimate the magnitude, trend, and effect of liver disease in Colombia as well as to explore its association with socioeconomic and behavioral factors. These results can be used by decision‐makers to plan and implement actions in public health (i.e. strengthening healthy living habits, intervening based on risk factors, promoting protective factors, paying closer attention in reference centers, and developing effective, fair, equitable, inclusive public policies for the access and distribution of health resources). These results can also help to reduce costs for the health system as well as decrease the loss of productivity for the country, understanding that liver disease is mostly preventable and, in minor proportions, curable.^35^

### *Limitations and bias*

Despite the great efforts of the Ministry of Health to improve and strengthen the Health Information System, cases of liver disease are still underreported. This is a limitation that worsens in departments (states) that have weaker health information systems and/or a deficit of specialists for the treatment of liver disease as well as technology that can make an early diagnosis.

There are no records of population coverage of hepatitis B vaccination for patients over 1 year of age, so it is not possible to determine the relationship between immunization and prevalence. It was reported that 57% of cirrhosis was attributable to either HBV (30%) or HCV (27%) globally,^36^ but we did not have information regarding chronic liver disease associated with hepatitis B or C. Since the earlier the infection of HBV heightens the risk of chronic liver disease,^37^ it can be anticipated that in the future, chronic liver disease associated with HBV will decrease given the introduction of the HBV vaccine in the EPI program in 1993. The number of cases of hepatitis B during the period of the study (2009–2016) decreased in Colombia. On the other hand, the number of cases of hepatitis C have increased in the last 6 years^38^ because reporting has improved because MOH is currently providing the proper treatment for the disease. However, an association between hepatitis C and chronic liver disease has not been established in studies reported from the country.

Because we used administrative data (RIPS that come to MOH from heath care providers), there may be misclassification due to either underreporting or the fact that some patients with liver disease may have been reported under a different diagnosis due to reports coming from institutions or doctors' offices with a low level of care.

The information bias was controlled with the definition of the diagnoses of interest according to the tenth version of the International Classification of Diseases (ICD‐10) and its definition of categories in order to cover all the possibilities of diagnoses related to the hepatic disease event.

## Supporting information


**Figure S1** Geographic distribution of viral infections Colombia 2009 and 2016.

**Figure S2** Proportion of liver cirrhosis 2016 and per capita alcohol sales (liters) by department in 2012.

**Table S1** Summary of data, liver disease by category and year.

## References

[1] Lim Y‐S , Kim WR . The global impact of hepatic fibrosis and end‐stage liver disease. Clin. Liver Dis. 2008; 12: 733–46. 18984463 10.1016/j.cld.2008.07.007

[2] Alves de Mattos A , Alsina Nader L . Epidemiología de las enfermedades hepáticas en Latinoamérica In: Méndez‐Sánchez N, Uribe M (eds). Hepatología Conceptos básicos y clínicos. México, DF: McGraw‐Hill, 2016 Available from URL: https://accessmedicina.mhmedical.com/content.aspx?bookid=1804&sectionid=123173377.

[3] Wang F , Fan J , Zhang Z , Gao B , Wang H . The global burden of liver disease: the major impact of China. Hepatology. 2014; 60: 2099–108. 25164003 10.1002/hep.27406 PMC4867229

[4] Guy J , Peters MG . Liver disease in women: the influence of gender on epidemiology, natural history, and patient outcomes. Gastroenterol. Hepatol. 2013; 9: 633–9. PMC3992057 24764777

[5] Mukherjee PS , Vishnubhatla S , Amarapurkar DN *et al* Etiology and mode of presentation of chronic liver diseases in India: a multi centric study. PLoS One. 2017; 12: 1–13. 10.1371/journal.pone.0187033 PMC5658106 29073197

[6] Beltran O , Galindo A , Mendoza Y *et al* Guía de práctica clínica para la enfermedad hepática grasa no alcohólica. Rev. Col. Gastroenterol. 2015; 30: 89–96.

[7] Bustíos D , Román Z . Características Epidemiológicas y Clínicas de la Cirrosis Hepática en la Unidad de Hígado del HNERM Es‐Salud. Rev. Gastroenterol. del Perú. 2007; 27: 238–45. 17934537

[8] Organization WH . Global hepatitis report, 2017. 2017; pp. 1–83. Cited 20 Feb 2018. Available from URL: https://www.who.int/hepatitis/publications/global-hepatitis-report2017/en/

[9] Ministerio de Salud y Protección Social . Cobertura de vacunación Colombia 2012–2017. 2017 Cited 14 Feb 2018. Available from URL: https://www.minsalud.gov.co/salud/publica/Vacunacion/Paginas/pai.aspx

[10] Cao G , Yi T , Liu Q , Wang M , Tang S . Alcohol consumption and risk of fatty liver disease: a meta‐analysis. PeerJ. 2016; 4: e2633. 27812428 10.7717/peerj.2633 PMC5088606

[11] LoConte NK , Brewster AM , Kaur JS , Merrill JK , Alberg AJ . Alcohol and cancer: a statement of the American Society of Clinical Oncology. J. Clin. Oncol. 2018; 36: 83–93. 29112463 10.1200/JCO.2017.76.1155

[12] Simpson R , Hermon C , Liu B *et al* Alcohol drinking patterns and liver cirrhosis risk: analysis of the prospective UK Million Women Study. Lancet. 2018; 4: e41–8. 30472032 10.1016/S2468-2667(18)30230-5 PMC6323353

[13] Askgaard G , Grønbæk M , Kjær MS , Tjønneland A , Tolstrup JS . Alcohol drinking pattern and risk of alcoholic liver cirrhosis: a prospective cohort study. J. Hepatol. 2015; 62: 1061–7. 25634330 10.1016/j.jhep.2014.12.005

[14] Bosetti C , Levi F , Lucchini F , Zatonski WA , Negri E , La Vecchia C . Worldwide mortality from cirrhosis: an update to 2002. J. Hepatol. 2007; 46: 827–39. 17336419 10.1016/j.jhep.2007.01.025

[15] Rehm J , Samokhvalov AV , Shield KD . Global burden of alcoholic liver diseases. J. Hepatol. 2013; 59: 160–8. 23511777 10.1016/j.jhep.2013.03.007

[16] Collins SE . Associations between socioeconomic factors and alcohol outcomes. Alcohol Res. 2016; 38: 83–94. 27159815 10.35946/arcr.v38.1.11 PMC4872618

[17] Pontiles de Sánchez M , Morón de Salim A , Rodríguez de Perdomo H , Perdomo Oramas G . Prevalencia de la enfermedad de hígado graso no alcohólico (EHGNA) en una población de niños obesos en Valencia, Venezuela. Arch. Latinoam. Nutr. 2014; 64: 73–82. 25799683

[18] Zhang KY , Tung BY , Kowdley KV . Liver transplantation for metabolic liver diseases. Clin. Liver Dis. 2007; 11: 265–81. 17606206 10.1016/j.cld.2007.04.002

[19] Guy J , Yee H . Health disparities in liver disease. Hepatology. 2010; 50: 309–13. 10.1002/hep.22942 PMC2705477 19554619

[20] Sistema Integral de Informacion de la Protección Social, Ministerio de Salud y Protección Social. Available from URL: http://www.sispro.gov.co/. [Cited in 7 March 2018].

[21] Bennett NG , Horiuchi S . Mortality estimation from registered deaths in less developed countries. Demography. 1984; 21: 217–33. 6734860

[22] Departamento Administrativo Nacional de Estadística (DANE) . Cited 8 Oct 2018. Available from URL: https://www.dane.gov.co/index.php/en/

[23] StataCorp . Stata statistical software: release 14. College Station, TX: StataCorp LP, 2015.

[24] Joinpoint Regression Program, Version 4.6.0.0 – April 2018. Statistical Methodology and Applications Branch, Surveillance Research Program, National Cancer Institute.

[25] Epidat: programa para análisis epidemiolóxico de datos. Versión 4.2, xullo. 2016 Consellería de Sanidade, Xunta de Galicia, España; Organización Panamericana da saúde (OPS‐OMS); Universidade CES, Colombia. Available from URL: https://www.sergas.es/Saude-publica/EPIDAT-4-2. [Cited in 15 Jul 2018].

[26] Andrade V , Mosos JD , Polanía MJ , Pacheco B , Rosselli D , Yucumá D . Venta de alcohol y tasa de enfermedad hepática alcohólica por departamentos en Colombia. Rev. Colomb. Gastroenterol. 2015; 30: 407–11.

[27] IBM Corp . Released 2016. IBM SPSS Statistics for Windows, Version 24.0. Armonk, NY: IBM Corp.

[28] Escorcia E , Marrugo W . Caracterización epidemiológica y clínica de la cirrosis hepatica en un centro regional del caribe colombiano: clínica general del norte. Enero 2012 a marzo 2017. Universidad Libre seccional Barranquilla; 2017.

[29] Adler NE , Newman K . Socioeconomic disparities in health: pathways and policies. Health Aff. 2002; 21: 60–76. 10.1377/hlthaff.21.2.60 11900187

[30] Arroyave I , Burdorf A , Cardona D , Avendano M . Socioeconomic inequalities in premature mortality in Colombia, 1998‐2007: The double burden of non‐communicable diseases and injuries. Prev. Med. 2014; 64: 41–7. 24674854 10.1016/j.ypmed.2014.03.018 PMC4067972

[31] Mathurin P , Bataller R . Trends in the management and burden of alcoholic liver disease. J. Hepatol. 2015; 62: S28–46. 10.1016/j.jhep.2015.03.006 PMC5013530 25920088

[32] Mokdad AA , Lopez AD , Shahraz S *et al* Liver cirrhosis mortality in 187 countries between 1980 and 2010: a systematic analysis. BMC Med. 2014; 12: 1–24. 10.1186/s12916-014-0145-y PMC4169640 25242656

[33] Neff GW , Duncan CW , Schiff ER . The current economic burden of cirrhosis. Gastroenterol Hepatol. 2011; 7: 661–71. PMC3265008 22298959

[34] Ruhl C , Sayer B , Byrd‐Holt D , Brown DM . Costs of digestive diseases In: NIH Publication (ed.). Burden of Digestive Diseases in the United States Report. National Institute of Diabetes and Digestive and Kidney Diseases. National Institutes of Health; Washington: 2008; 137–43.

[35] Esser MB , Hedden SL , Kanny D , Brewer RD , Gfroerer JCNT . Prevalence of alcohol dependence among US adult drinkers, 2009–2011. Prev. Chronic. Dis. 2014; 11: 1–11. 10.5888/pcd11.140329 PMC4241371 25412029

[36] Perz JF , Armstrong GL , Farrington LA , Hutin YJF , Bell BP . The contributions of hepatitis B virus and hepatitis C virus infections to cirrhosis and primary liver cancer worldwide. J. Hepatol. 2006; 45: 529–38. 16879891 10.1016/j.jhep.2006.05.013

[37] Alegría S . Hepatitis crónica. Rev. Child Pediatr. 2002; 73: 176–80.

[38] Ministerio de Salud y Protección Social, Instituto Nacional de Salud. Boletín Epidemiológico Semanal, Semana epidemiológica 28 (7 al 13 de julio de 2019). 2019 Available from URL: https://www.ins.gov.co/buscador‐eventos/BoletinEpidemiologico/2019%20Bolet%C3%ADn%20epidemiol%C3%B3gico%20semana%2028.pdf. [Cited in 5 Dec 2019].

