# Supplementary figures and images for: Prevalence of liver disease in Colombia between 2009 and 2016
Source: JGH Open. 2020 Feb 19;4(4):603–10. doi: 10.1002/jgh3.12300 (PMC7411567; doi:10.1002/jgh3.12300)

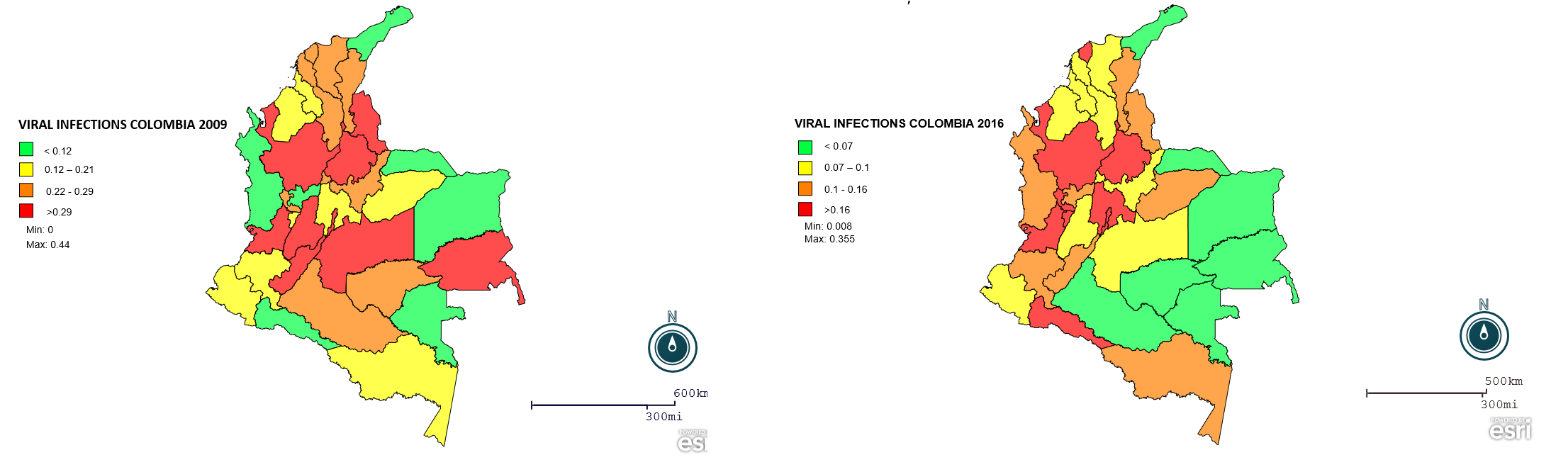

Supplement: Supplementary file 1 — Figure S1 Geographic distribution of viral infections Colombia 2009 and 2016. [file JGH3-4-603-s001.tiff]

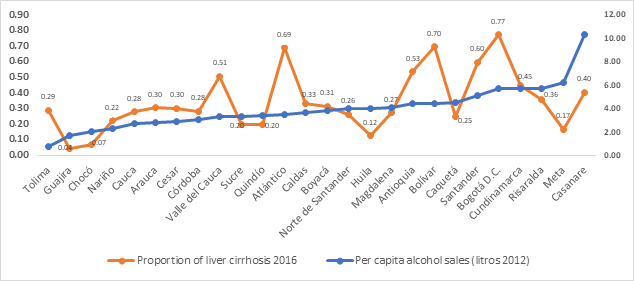

Supplement: Supplementary file 2 — Figure S2 Proportion of liver cirrhosis 2016 and per capita alcohol sales (liters) by department in 2012. [file JGH3-4-603-s002.tiff]
