# Supplementary material for: Prevalence of liver disease in Colombia between 2009 and 2016
Source: JGH Open. 2020 Feb 19;4(4):603–10. doi: 10.1002/jgh3.12300 (PMC7411567; doi:10.1002/jgh3.12300)
Supplement: Supplementary file 3 — Table S1 Summary of data, liver disease by category and year. [file JGH3-4-603-s003.docx]

**Supplementary table 1:** Summary of data, liver disease by category and year.

|  |  | **2009** | **2010** | **2011** | **2012** | **2013** | **2014** | **2015** | **2016** |
| --- | --- | --- | --- | --- | --- | --- | --- | --- | --- |
| **Hepatic disease** | **Cases** | 55,649 | 55,767 | 71,825 | 86,394 | 91,400 | 108,242 | 91,380 | 89,231 |
|  | **% Male** | 44% | 43% | 43% | 43% | 42% | 41% | 40% | 40% |
|  | **% Female** | 56% | 57% | 57% | 57% | 58% | 59% | 60% | 60% |
|  | **National Rate*** | 1.213 | 1.184 | 1.487 | 1.749 | 1.811 | 2.086 | 1.725 | 1.640 |
|  | **Minimum*** | 0.022 | 0.034 | 0.144 | 0.169 | 0.188 | 0.338 | 0.059 | 0.117 |
|  | **Maximum*** | 2.864 | 1.950 | 2.373 | 2.734 | 3.641 | 4.010 | 2.628 | 2.833 |
| **Cirrhosis** | **Cases** | 9,650 | 11,385 | 13,315 | 16,699 | 18,056 | 23,384 | 21,717 | 22,588 |
|  | **% Male** | 48% | 47% | 46% | 45% | 44% | 43% | 42% | 41% |
|  | **% Female** | 52% | 53% | 54% | 55% | 56% | 57% | 58% | 59% |
|  | **National Rate*** | 0.205 | 0.235 | 0.267 | 0.326 | 0.343 | 0.434 | 0.394 | 0.399 |
|  | **Minimum*** | 0.000 | 0.000 | 0.019 | 0.022 | 0.000 | 0.039 | 0.000 | 0.031 |
|  | **Maximum*** | 0.956 | 0.958 | 0.529 | 0.605 | 0.599 | 0.724 | 0.646 | 0.771 |
| **Congenital and of birth** | **Cases** | 642 | 682 | 910 | 1,222 | 1,228 | 1,282 | 1,589 | 1,410 |
|  | **% Male** | 28% | 27% | 26% | 24% | 24% | 23% | 24% | 22% |
|  | **% Female** | 72% | 73% | 74% | 76% | 76% | 77% | 76% | 78% |
|  | **National Rate*** | 0.014 | 0.014 | 0.019 | 0.024 | 0.025 | 0.030 | 0.026 | 0.025 |
|  | **Minimum*** | 0.000 | 0.000 | 0.000 | 0.000 | 0.000 | 0.000 | 0.000 | 0.000 |
|  | **Maximum*** | 0.040 | 0.027 | 0.032 | 0.044 | 0.044 | 0.052 | 0.043 | 0.051 |
| **Portal hypertension** | **Cases** | 558 | 552 | 751 | 816 | 808 | 953 | 779 | 854 |
|  | **% Male** | 48% | 45% | 46% | 45% | 46% | 43% | 44% | 48% |
|  | **% Female** | 52% | 55% | 54% | 55% | 54% | 57% | 56% | 52% |
|  | **National Rate*** | 0.012 | 0.012 | 0.015 | 0.016 | 0.016 | 0.018 | 0.015 | 0.016 |
|  | **Minimum*** | 0.000 | 0.000 | 0.000 | 0.000 | 0.000 | 0.000 | 0.000 | 0.000 |
|  | **Maximum*** | 0.116 | 0.035 | 0.048 | 0.029 | 0.030 | 0.047 | 0.033 | 0.054 |
| **Pregnancy** | **Cases** | 133 | 125 | 120 | 172 | 197 | 286 | 229 | 167 |
|  | **% Male** | 0% | 0% | 0% | 0% | 0% | 0% | 0% | 0% |
|  | **% Female** | 100% | 100% | 100% | 100% | 100% | 100% | 100% | 100% |
|  | **National Rate*** | 0.003 | 0.003 | 0.003 | 0.004 | 0.004 | 0.006 | 0.005 | 0.003 |
|  | **Minimum*** | 0.000 | 0.000 | 0.000 | 0.000 | 0.000 | 0.000 | 0.000 | 0.000 |
|  | **Maximum*** | 0.056 | 0.035 | 0.009 | 0.072 | 0.025 | 0.021 | 0.021 | 0.010 |
| **Non-cirrhotic alcoholic liver disease** | **Cases** | 861 | 1,075 | 1,272 | 1,286 | 1,412 | 2,056 | 1,551 | 1,109 |
|  | **% Male** | 69% | 64% | 65% | 62% | 57% | 56% | 57% | 57% |
|  | **% Female** | 31% | 36% | 35% | 38% | 43% | 44% | 42% | 43% |
|  | **National Rate*** | 0.019 | 0.023 | 0.026 | 0.026 | 0.028 | 0.039 | 0.029 | 0.020 |
|  | **Minimum*** | 0.000 | 0.000 | 0.000 | 0.000 | 0.000 | 0.000 | 0.000 | 0.000 |
|  | **Maximum*** | 0.046 | 0.052 | 0.064 | 0.046 | 0.059 | 0.084 | 0.048 | 0.043 |
| **Inflammatory** | **Cases** | 1,533 | 1,797 | 2,359 | 2,961 | 3,066 | 3,668 | 3,149 | 3,137 |
|  | **% Male** | 3% | 4% | 5% | 6% | 6% | 7% | 6% | 6% |
|  | **% Female** | 45% | 43% | 44% | 42% | 41% | 41% | 39% | 38% |
|  | **National Rate*** | 0.549 | 0.572 | 0.564 | 0.583 | 0.587 | 0.590 | 0.607 | 0.621 |
|  | **Minimum*** | 0.000 | 0.000 | 0.000 | 0.000 | 0.000 | 0.000 | 0.000 | 0.000 |
|  | **Maximum*** | 0.265 | 0.267 | 0.301 | 0.090 | 0.377 | 0.518 | 0.516 | 0.643 |
| **Metabolic** | **Cases** | 28,743 | 28,105 | 38,205 | 48,407 | 49,482 | 62,063 | 51,371 | 50,836 |
|  | **% Male** | 39% | 38% | 39% | 39% | 39% | 38% | 37% | 37% |
|  | **% Female** | 61% | 62% | 61% | 61% | 61% | 62% | 63% | 63% |
|  | **National Rate*** | 0.620 | 0.593 | 0.786 | 0.973 | 0.973 | 1.192 | 0.974 | 0.938 |
|  | **Minimum*** | 0.000 | 0.000 | 0.000 | 0.021 | 0.042 | 0.037 | 0.000 | 0.000 |
|  | **Maximum*** | 7.149 | 6.704 | 7.140 | 1.749 | 9.224 | 11.272 | 9.574 | 12.809 |
| **Toxic** | **Cases** | 1,360 | 1,529 | 1,909 | 2,090 | 2,260 | 2,979 | 2,765 | 2,316 |
|  | **% Male** | 43% | 46% | 44% | 42% | 37% | 38% | 34% | 36% |
|  | **% Female** | 57% | 54% | 56% | 58% | 63% | 62% | 66% | 64% |
|  | **National Rate*** | 0.030 | 0.033 | 0.040 | 0.043 | 0.045 | 0.058 | 0.053 | 0.044 |
|  | **Minimum*** | 0.000 | 0.000 | 0.000 | 0.000 | 0.000 | 0.000 | 0.000 | 0.000 |
|  | **Maximum*** | 0.037 | 0.048 | 0.065 | 0.076 | 0.070 | 0.106 | 0.096 | 0.078 |
| **Liver tumors** | **Cases** | 1,374 | 1,836 | 2,141 | 2,197 | 4,844 | 2,645 | 2,386 | 2,132 |
|  | **% Male** | 45% | 44% | 46% | 47% | 39% | 44% | 46% | 44% |
|  | **% Female** | 55% | 56% | 54% | 53% | 60% | 56% | 54% | 56% |
|  | **National Rate*** | 0.029 | 0.038 | 0.043 | 0.042 | 0.095 | 0.048 | 0.043 | 0.037 |
|  | **Minimum*** | 0.000 | 0.000 | 0.000 | 0.000 | 0.000 | 0.000 | 0.000 | 0.000 |
|  | **Maximum*** | 0.052 | 0.072 | 0.062 | 0.103 | 0.159 | 0.068 | 0.053 | 0.059 |
| **Vascular** | **Cases** | 243 | 190 | 212 | 229 | 220 | 323 | 257 | 187 |
|  | **% Male** | 37% | 38% | 38% | 36% | 36% | 37% | 37% | 40% |
|  | **% Female** | 63% | 62% | 62% | 64% | 64% | 63% | 63% | 60% |
|  | **National Rate*** | 0.005 | 0.004 | 0.004 | 0.005 | 0.005 | 0.007 | 0.005 | 0.004 |
|  | **Minimum*** | 0.000 | 0.000 | 0.000 | 0.000 | 0.000 | 0.000 | 0.000 | 0.000 |
|  | **Maximum*** | 0.010 | 0.007 | 0.009 | 0.017 | 0.025 | 0.036 | 0.010 | 0.010 |
| **Viral infections** | **Cases** | 12,124 | 10,214 | 12,908 | 12,797 | 12,564 | 11,969 | 8,678 | 7,439 |
|  | **% Male** | 53% | 51% | 50% | 52% | 51% | 51% | 49% | 49% |
|  | **% Female** | 47% | 49% | 50% | 48% | 49% | 49% | 51% | 51% |
|  | **National Rate*** | 0.277 | 0.228 | 0.283 | 0.279 | 0.269 | 0.248 | 0.174 | 0.145 |
|  | **Minimum*** | 0.000 | 0.000 | 0.057 | 0.056 | 0.079 | 0.068 | 0.000 | 0.008 |
|  | **Maximum*** | 0.448 | 0.338 | 1.318 | 0.538 | 1.019 | 0.418 | 0.309 | 0.355 |

* The national and departmental rates (minimum and maximum) are presented adjusted x 1,000 inhabitants.
